# BanglaVeg: A curated vegetable image dataset from Bangladesh for precision agriculture

**DOI:** 10.1016/j.dib.2025.111441

**Published:** 2025-03-04

**Authors:** Md Jobayer Ahmed, Ratu Saha, Arpon Kishore Dutta, Mayen Uddin Mojumdar, Narayan Ranjan Chakraborty

**Affiliations:** Department of Computer Science and Engineering, Daffodil International University, Daffodil Smart City, Birulia, Dhaka 1216, Bangladesh

**Keywords:** Classification of vegetables, Image recognition, Machine learning, Bangladeshi vegetables, Images dataset, Deep learning

## Abstract

Vegetables are one of the most essential parts of the agricultural sector and the food supply chain; therefore, the identification and categorization of vegetable types require effective strategies. In this paper, we introduce the Vegetable Image Dataset, which is a meticulously developed collection of 4319 images representing 12 different vegetable species native to Bangladesh, including Potato, Onion, Green Chili, Garlic, Radish, Bean, Ladies Finger, Cucumber, Bitter Melon, Brinjal (Eggplant), Tomato, Pointed Gourd. The dataset contains images taken in natural environments, including local markets, agricultural fields, and homes, using phone cameras to represent real-world conditions better. All photos have undergone background removal and annotation to highlight features such as shape, texture, and color, thus making it a handy resource for deep-learning projects. Developed primarily for developing convolutional neural network (CNN) models, this dataset allows for the automatic identification and classification of vegetables for various applications. Applications range from improving the supply chain for agriculture to allowing instantaneous detection of vegetables in kitchens or marketplaces and increasing the efficiency of automation for sorting and packaging. With its unique characteristic of Bangladeshi vegetables, this dataset provides the valuable resource needed for improving agricultural practices using AI-driven ways and fostering further developments of technologies in underserved communities.

Specifications Table

**Table:** 

Subject	Agricultural and Biological Sciences.
Specific subject area	Computer Vision, Image Classification, Machine learning, Image Recognition, Deep Learning, Image Processing,
Type of data	Images (total of 4319 Raw images), Table.
Data collection	Field surveys were conducted from September to November 2024 under the supervision of agricultural experts in different environmental conditions. The dataset contains 4319 high-resolution images representing vegetables from 12 different classes collected from several places in Bangladesh, such as farmhouses, local markets, and houses. This significant accumulation of visual features is necessary to train and test machine learning algorithms devoted to vegetable classification. The images captured by: (i) iPhone 15, (ii) Xiaomi Poco F4, and (iii) Redmi Note 10 Pro
Data source location	Bangladesh (the particular capture sites were not tagged with geographical coordinates).
Data accessibility	Repository name: Mendeley Data [13] Data identification number: 10.17632/b9rvg4f2st.4 Direct URL to data: https://data.mendeley.com/datasets/b9rvg4f2st/4

## 1 Value of the Data

• Vegetable Image Dataset: This includes 4319 high-resolution images of 12 different classes of vegetables taken in various locations within Bangladesh, thus offering different regional diversity for vegetable classification with accurate context.
• Better Classification Models: This dataset is essential in training and testing deep learning models, especially Convolutional Neural Networks (CNNs), to improve algorithms for vegetable identification and classification using features such as shape, size, color, and texture.
• Varied Uses: It enables automatic vegetable recognition in agricultural and commercial settings. It also allows the digitization of food supply chains, including sorting and distribution processes. Finally, it supports quality assurance in the packaging and processing of vegetables.
• Wide Range of Applications: Helps find vegetables in automated kitchen facilities and supermarket systems. Improves agricultural automation, such as harvesting crops and sorting produce. Ensures the maintenance of vegetable quality all along the supply chain.

## 2 Background

Vegetables play a critical and vital role in the cuisine, agriculture, and commerce of the whole world, especially in Bangladesh, where they are rooted deep into people's everyday lives and cultural practices of people. Because of this fact, and as the demand for fresh produce is ever-increasing at a robust and consistent rate locally and internationally, many problems remain in efficiently identifying and classifying vegetables due to various shapes, sizes, textures, and colors. Traditional methods of sorting and grading used for so many years are often subjective, laborious, and inefficient, hence demanding technological intervention to improve them.

There has been a rapid and tremendous stride in artificial intelligence, more precisely in convolutional neural networks (CNNs), which brought a staggering transformation in image-based classification systems. Nevertheless, it needs to be noted that overall effectiveness and efficiency in such advanced AI models remain hugely dependent on the availability of large and diverse datasets. With such an essential need in mind, the Vegetable Image Dataset was created, where images were represented, corresponding to 4319 well-annotated images. The data collection occurred in several contexts within Bangladesh, ensuring that it accounts for the other variations encountered in real-life settings [8]. Images were collected from natural scenarios in farms, busy markets, and houses to ensure the diversity of real-world conditions and context relevance as required for model training and evaluation.

This dataset is of utmost importance and is pivotal in developing and advancing AI-driven systems designed to automate various tasks in agriculture, food processing, and supply chain management. With great importance, such can significantly minimize human intervention in these processes, potentially increasing overall efficiency and consistently ensuring quality, thereby effectively streamlining operations in sorting and packaging. Moreover, this dataset makes it possible to reliably and quickly identify vegetables, hence contributing to achieving speedier production cycles and, at the same time, ensuring a reduction in wastage throughout the whole process.

This also forms an essential opportunity for interdisciplinary research collaborations in this fascinating field of integrating computer vision with novel sensory technologies to analyze the different features of vegetables holistically and precisely. So, this dataset gives a perfect foundation for new applications related to agriculture and food systems and marks a landmark, impactful advance toward better automation and efficiency in those vital sectors responsible for our supply of food and improvement in agricultural practices.

## 3 Data Description

The vegetable market plays a significant role in the agricultural sector, contributing a substantial share to overall profits [[1], [2], [3], [4], [5]]. This data set includes a diverse collection of 4319 raw images of different vegetables, divided into 12 distinct categories and collected from multiple sources like markets, houses, and shops all over Bangladesh. The vegetable types include pointed gourd, bitter melon, brinjal (eggplant), etc., native to the region. They are all well-organized in separate folders for easy access. Using computer vision and deep learning methodologies, the vegetables are systematically classified on apparent features such as shape, size, and color. This enhances efficiency and reliability during the process [6,7].

Photos were taken from various angles, in natural and artificial light conditions, to ensure a wide range of diversity and quality [9]. These images represent realistic scenarios, showing vegetables in their natural settings, thus providing an extensive collection of visual features necessary for training and testing machine learning algorithms.

The dataset contains only images in JPG format, which have been resized to maintain consistent quality while improving storage capacity and processing efficiency. All directories are named according to their respective vegetable category, making them easily recognizable and usable. This dataset is a vital resource for developing computer vision models, machine learning algorithms, and applications in agricultural informatics, not to mention contributing to culinary research and supply chain optimization in the food sector.

Like all images in the data, this image is in JPG format: it allows for maintaining higher image qualities, provides cross-platform compatibility, and keeps file sizes small for better manageability. The dataset is also available as a zip, making downloading and integration into different applications easy for users. The data set has twelve categories, as shown in the Table 2 below:

**Table 1:** Comparison with existing datasets.

SL	Vegetable	Our Dataset	Md. T. Ahmad Bappy et al. [15]	Mostafa El-Ghoul, S.S. Abu-Naser, et al. [14]	W. Wang, A. Zhu, et al. [16]	K. Guo, et al. [17]
1	Potato	365	X	1000	X	✔ (Not-
						Mentioned)
2	Onion	357	X	X	682	X
3	Green Chili	497	X	X	X	X
4	Garlic	349	X	X	X	X
5	Radish	310	150	1000	X	X
6	Bean	454	150	1000	X	X
7	Ladies Finger	308	150	X	X	X
8	Cucumber	342	X	1000	682	X
9	Pointed Gourd	329	150	X	X	X
10	Bitter Melon	306	150	1000	X	X
11	Brinjal	373	X	1000	X	X
12	Tomato	329	150	1000	682	✔ (Not-
						Mentioned)

Note: (X) means data not available.

(✔) means data is available, but the dataset number was not mentioned.

Table 1 provides a detailed comparison between my dataset and existing datasets.

**Table 2:** Provides the amount of analysis of images along with their corresponding counts and scientific names. **Dataset Summary**:

SI No	Class	Description	Image	Quantity
1	Potato (Solanum tuberosum)	Potatoes are tubers that grow underground and are part of the nightshade family. Their shape, size, and skin color may vary greatly: they can be white, yellow, red, purple, or countless other shades. The interior usually is white or yellow with a soft, starchy texture.		365
2	Onion (Allium cepa)	Onions are round and have layers. They come in different varieties: red, yellow, and white. They have a strong, sharp taste when raw but become softer and sweeter when cooked.		357
3	Green Chili (Capsicum annuum)	Green chillies are thin, narrow, elongated peppers with a pungent and hot taste. The intensity may vary depending on variety and maturity.		497
4	Garlic (Allium sativum)	Garlic is a vegetable with a bulb. The bulb contains many cloves covered by a thin white or purple skin. Garlic has a pungent odor and flavor.		349
5	Radish (Raphanus sativus)	Radishes are crunchy, sweet vegetables growing underground. They taste hot. Radishes come in different sizes and colors: red, white, and purple.		310
6	Bean (Phaseolus vulgaris)	Long, slender bean pods contain edible seeds. One popular type is green beans, which are harvested while tender.		454
7	Ladies Finger (Abelmoschus esculentus)	It is known as okra, a green, lengthy vegetable with a slimy interior and edible seeds.		308
8	Cucumber (Cucumis sativus)	Cucumbers are a fresh and juicy vegetable. They are crunchy in texture with smooth or slightly bumpy green skins and light-colored flesh.		342
9	Pointed Gourd (Trichosanthes dioica)	Pointed Gourd, or “parwal,” is an extended green vegetable with a faint flavor. It is used in Indian and Bangladeshi cooking.		329
10	Bitter Melon (Momordica charantia)	Bitter melon is a green, bumpy vegetable that tastes very bitter. It's also called "bitter gourd ".		306
11	Brinjal (Solanum melongena)	Brinjal, or eggplant, comes in different sizes, shapes, and colors, such as deep purple and white or black.		373
12	Tomato (Solanum lycopersicum)	Tomatoes are juicy fruits that are used in the kitchen as vegetables. They are red when ripe and have a slightly tangy, sweet taste.		329
	Total			4319

## 4 Experimental Design, Materials and Methods

### 4.1 Experimental design

The dataset for this study was collected from numerous vegetable markets, houses, and shops in Bangladesh from September 2024 to February 2025. The was created using five steps: finding vegetable sources, choosing vegetables, image acquisition, processing images, and storing the dataset.

The Vegetable Image Dataset for classification was carefully created to respond to the need for extensive collections of vegetable images from specific areas. The main aim of this dataset is to help machine learning models, especially CNNs, automatically sort vegetables based on their visible features like shape, size, color, and texture. The following section describes the steps taken in creating the dataset, including how the images of vegetables were collected, captured, and prepared for use by machine learning models. I approached the process by finding locations with different vegetable types, including local markets, houses, and grocery stores.

The selection of vegetables ensures that all 12 categories are well represented and, thus, show considerable natural variability. Images were photographed with high-quality smartphone cameras under different setups—natural and artificial light—to keep the variety and quality of visual features in these images. After taking the photos, they were edited to look similar and easy to apply by making them smaller, removing the background, and so on. Lastly, the modified photos were organized and saved into clean folders to be easily accessible and ready for quick use in machine learning tasks and academic research. The process ascertains that this dataset represents the diverse Bangladeshi vegetables and is valuable for advancing computer vision and agricultural informatics.

**Fig. 1:**
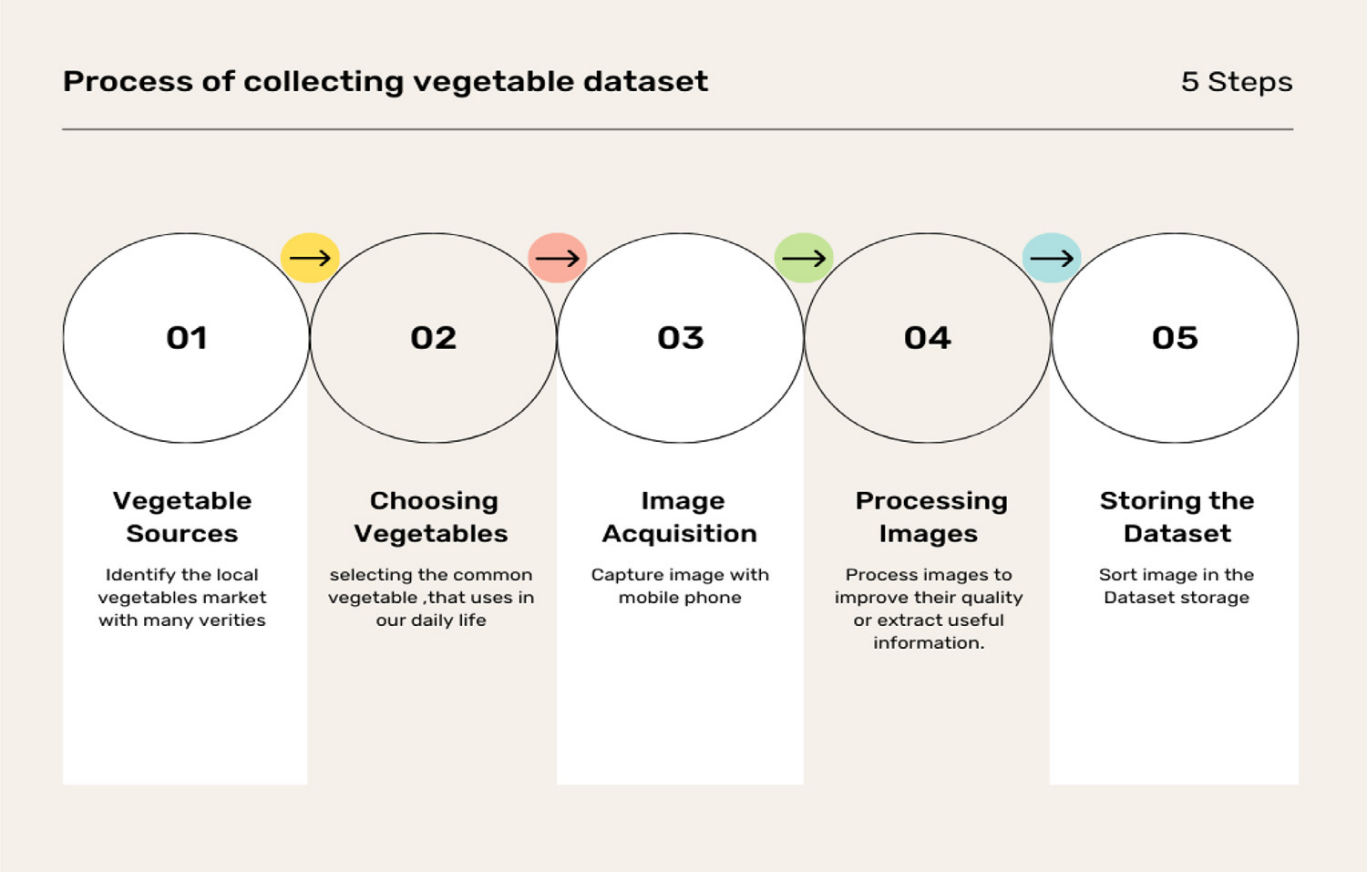
Step-by-step progress: Gathering the vegetable dataset.

Fig. 1 shows the step-by-step processing of collecting vegetable images.

### 4.2 Finding vegetable sources

The first step was finding sources for vegetable samples to ensure the dataset had variations arising from differences in how the vegetables were grown, the environmental conditions, and storage. Accordingly, vegetables were collected from various places in Bangladesh to represent real-world conditions and variations.

Grocers: Most vegetables bought in grocery stores are packaged and stored. These vegetables undergo many handling, packaging, and storage procedures that may change their appearance, texture, and color. These examples illustrate those differences due to commercial practices.

Families: Samples from home settings give information on vegetables kept under usual household conditions. Being exposed to air, differences in humidity levels, and how long they are stored can cause slight changes in the physical features of the vegetables, like color, texture, and freshness.

Markets: Markets are prominent places to get fresh fruits and vegetables. Vegetables from these places usually show signs of little storage and are more likely to be recently picked crops. These samples show natural differences because of the way they are handled right after being harvested. The dataset contains these differences from various sources, ensuring it fully displays the different factors affecting the appearance and quality of vegetables. This diversity makes the dataset strong and appropriate for training machine-learning models in farming, cooking, and industry.

### 4.3 Choosing vegetables

The next step in creating our dataset was choosing specific categories of vegetables to guarantee variety and suitability. For this dataset, 12 edible vegetables that are commercially used and produced in Bangladesh were selected. Such vegetables were chosen because they are utilized in local cuisines, are essential for the agricultural economy, and have specific physical characteristics appropriate for machine learning classification problems.

The selected vegetable categories include Potato, Onion, Green Chili, Garlic, Radish, Bean, Ladies Finger, Cucumber, Bitter Melon, Brinjal (Eggplant), Tomato, and Pointed gourd.

The vegetable crops considered in this study have been approved because they are common in Bangladeshi food culture. Numerous visual features, such as different forms, dimensions, colors, and textures, could make classification systems more complex. Such natural difficulties otherwise provide an avenue for building advanced machine-learning algorithms that can accurately classify different types of vegetables under other circumstances.

### 4.4 Image acquisition

Once the final selection of vegetable categories had been finalized, a second critical step was image shooting for the higher-quality dataset. Precise and consistent imaging is one of the essential requirements for deep learning applications. Therefore, special heed was paid when choosing equipment and conditions related to the environment where all images were shot—since it aimed to accurately and consistently depict the variety of visual features such as shape, texture, and color for each vegetable. This data set includes photos taken using iPhone 15, Xiaomi Poco F4, and Redmi Note 10 Pro.

1. iPhone 15: The iPhone 15 has an excellent camera system with better HDR and brilliant photography. Its 48MP primary camera takes great details, which helps to represent slight differences in the texture and color of vegetables. Moreover, its advanced photonic engine ensures that images look good even in low light, which helps when taking pictures of vegetables in places like indoor markets where the light is dim.
2. Xiaomi Poco F4: The Xiaomi Poco F4 comes with a 64MP triple-camera setup, which provides excellent detail and a range of colors. Its precise sensor and AI features helped to accurately show the detailed features of vegetables. It can take good pictures in both wide-angle and close-up modes, making it perfect for photographing different sizes of vegetables, from big gourds to small ones like green chilies.
3. Redmi Note 10 Pro: The Redmi Note 10 Pro has a 108MP primary camera that captures clear pictures. It accurately showed colors and detailing, making vegetables look real, even those with complex shapes like ridged gourds and leafy greens. In addition, its Night Mode helped take good pictures in low light, maintaining the quality of the images. In taking photos of these vegetables using different controlled settings, the dataset shows the diversity of Bangladeshi vegetables. This helps the dataset efficiently train strong machine-learning models with high precision.

Table 3 Offers a comprehensive overview of smartphone camera specifications, including phone model, detailed camera features, and the respective author.

**Table 3:** Specification of image acquisition device.

SI no	Author	Smart Phone	Camera specification
1	Md Jobayer Ahmed	iPhone 15	• 48MP Main: 26 mm, ƒ/1.6, sensor-shift stabilization, 100% Focus Pixels, supports 24MP and 48MP photos. • 12MP Ultra-Wide: 13 mm, ƒ/2.4, 120°FOV • 12MP 2x Telephoto: 52 mm, ƒ/1.6, sensor-shift stabilization, 100% Focus Pixels
2	Ratu Saha	Xiaomi Poco F4	• 64 MP, f/1.8, (wide) 1/2.0", 0.7µm, PDAF, OIS • 8 MP, f/2.2, 119° (ultrawide), 1/4.0", 1.12µm • 2 MP, f/2.4, (macro)
3	Arpon Kishore Dutta	Redmi Note 10 Pro	• 108MP,f/1.9,26mm(wide),1/1.52",0.7µm,PDAF • 8 MP, f/2.2, 118° (ultrawide), 1/4.0", 1.12µm

With these three phones—iPhone 15, Xiaomi Poco F4, and Redmi Note 10 Pro—many pictures of vegetables from varying angles and light settings were taken. By doing so, it is guaranteed that each vegetable, through the number of samples, was well-represented, making it less likely to change the variety of the dataset. Pictures were taken from overhead, side views, close-ups, and angles. It is essential to have other types of vegetables while training machine learning models, for it makes them learn more about each vegetable and perform better. In this manner, the lighting conditions were carefully managed to ensure the pictures were clear and consistent. Softbox lighting fixtures are used to reduce shadows and glare, which could interfere with the appearance of vegetables. Vegetables were placed on plain white sheets, ensuring nothing would distract from the primary subject in each image.

All images are saved in JPG format, which is common in deep learning since it balances file quality and size. The pictures were taken with the same level and aspect ratio, making them homogeneous across the whole dataset. Moreover, this format will ensure that opening, processing, and saving for storage will work quickly and efficiently—ideal characteristics for large datasets.

The attention to detail in this image capture process assures the quality and usability of the dataset for training accurate and reliable machine learning models.

### 4.5 Processing images

Once the images were captured, image classification became the next major step. Image classification is essential because it helps create a robust dataset for machine learning that links each image to its label corresponding to its type of vegetable. Categories including Potato, Tomato, and Brinjal were used to make the correct labels, ensuring the categories matched each image correctly.

To simplify the classification, images were placed in different folders, each representing a different type of vegetable. This folder setup made finding and using images much easier while training the machine-learning model. Each image was renamed in order within its category folder to keep things orderly and help manage the dataset well.

**Fig. 2:**
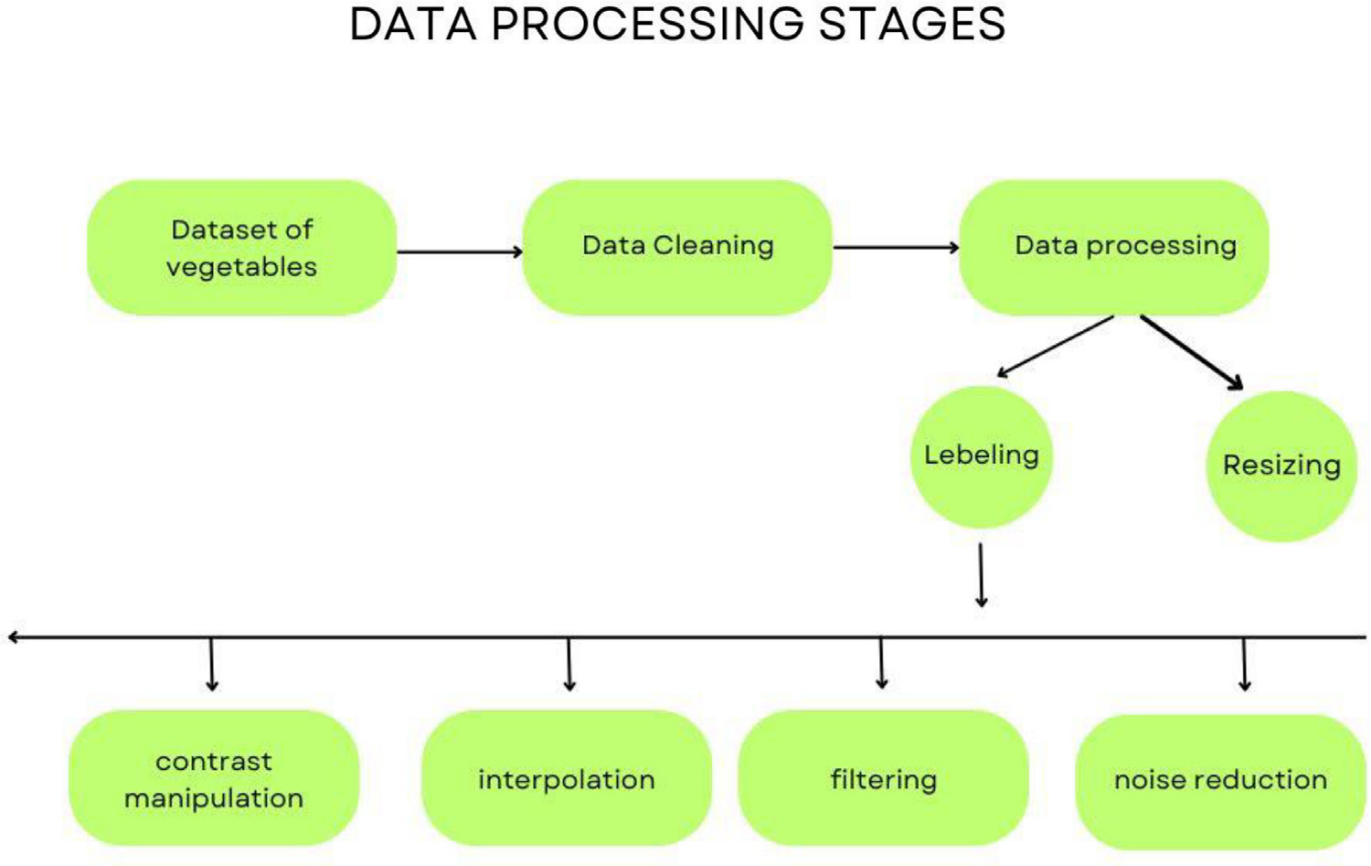
Data processing stages.

Fig. 2: Block diagram of the proposed approach for vegetable image processing. The proposed method includes data cleaning and preprocessing, image resizing to a standard size, and applying enhancements such as rotation, zooming, brightness variation, and shifting to increase the dataset size [10]. Such enhancement helped improve the dataset's quality and diversity, strengthening it for deep learning applications. The systematic labeling, organization, and preprocessing guaranteed consistency in the dataset, making it usable for creating accurate and efficient vegetable classification models.

### 4.6 Storing the dataset

Once the images were categorized and labeled, they were aggregated in a final vegetable dataset. Images in each category were arranged in specific folders with names representing the type of vegetable they stand for effortless navigation and an efficient structure in the dataset. For ease during the storage and transfer process, the dataset was archived in a ZIP file to make it easy to upload to cloud-based platforms and share via Mendeley Data.

The dataset's images are saved in JPG format because it works well with image processing and machine learning tools [11]. JPG provides good quality while keeping the file size small, which is excellent for large datasets. To maintain consistency, all images were resized to 720 × 1080p. Keeping a standard size for all images in a dataset is essential when using deep-learning models for training. It would ensure consistent input size during training and evaluation.

The ZIP compression format is used because it can reduce file size without losing quality. It packs many files into one archive, making storing and transferring data easier [12]. Its ability to work on different systems, manage large files, and provide encryption makes it even better at saving and sharing datasets like this.

**Fig. 3:**
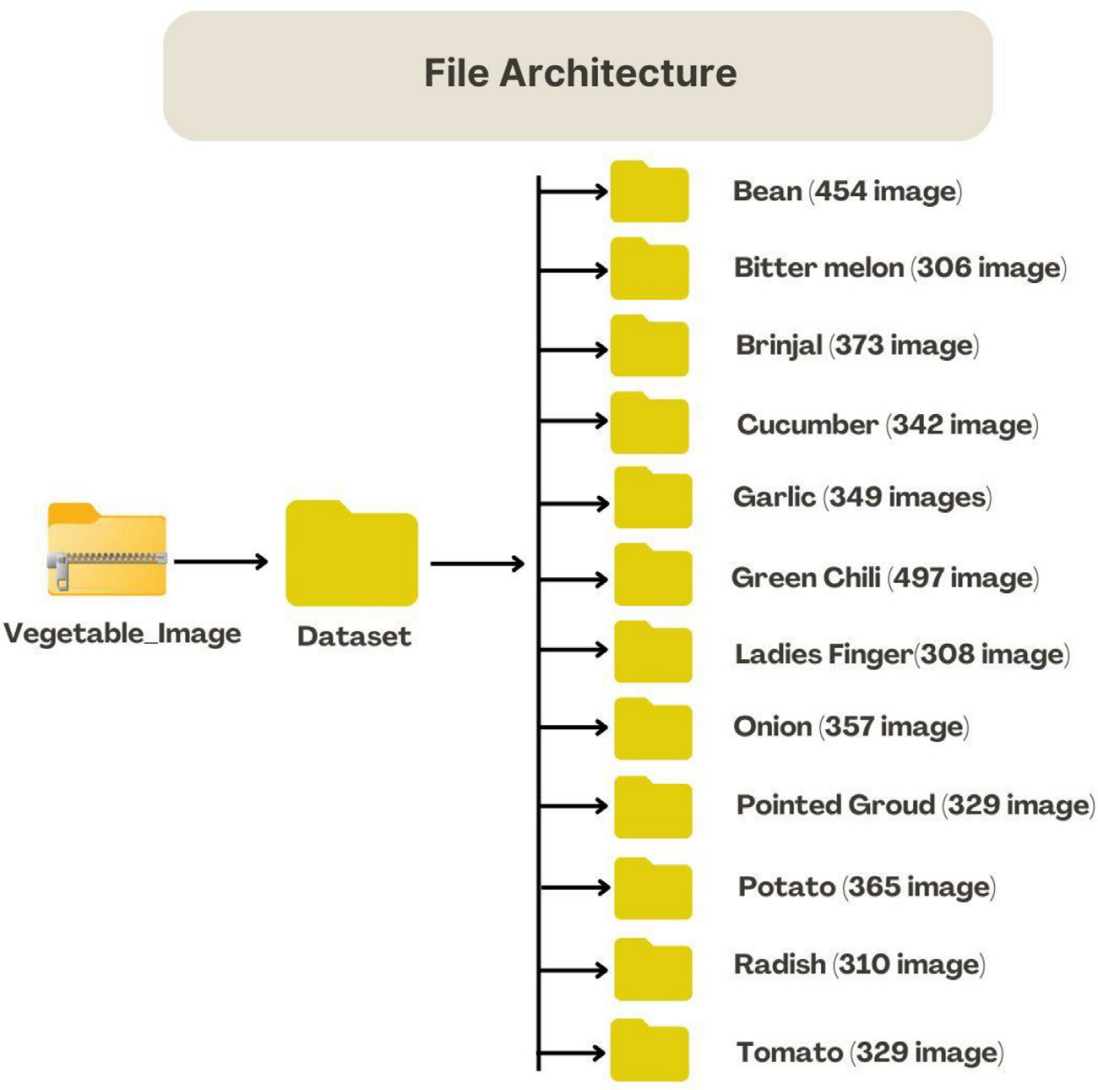
Step-by-step progress: Folder structure.

Fig. 3, shows the step-by-step process of storing the dataset, highlighting how the collected vegetable images are stored for further use.

## Limitations

The limits of the dataset mainly come from differences in lighting and environment, which can affect the model's performance in the most extreme instances. Second, we did not remove the background because we provided the raw Mendeley Data dataset. While this method keeps the backgrounds natural, it can also create problems for the adaptability of how well the model performs because of the differences in backgrounds.

## Ethics Statement

The authors state that this dataset adheres to ethical standards for publication in Data in Brief papers and does not include human subjects, animal experiments, or social media data.

## Credit Author Statement

**Md Jobayer Ahmed**: Conceptualization, Data curation, Validation, Methodology, Writing - Original Draft. **Ratu Saha**: Data curation, Resources. **Arpon Kishore Dutta**: Conceptualization, Formal analysis, Investigation, Data curation, Writing – review & editing. **Mayen Uddin Mojumdar:** Writing – review & editing. **Narayan Ranjan Chakraborty:** Writing – review & editing.

## Data Availability

Mendeley DataVegetable Image Dataset for Classification Models: A Bangladeshi Perspective (Original data)
